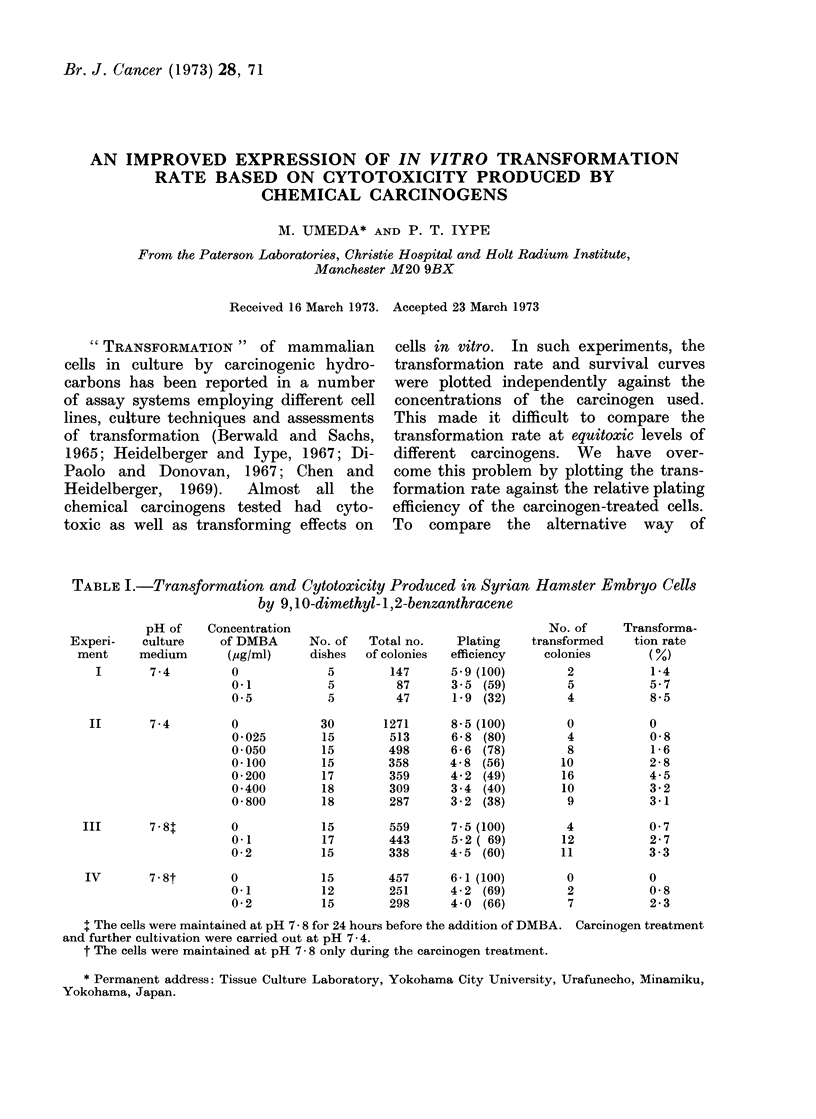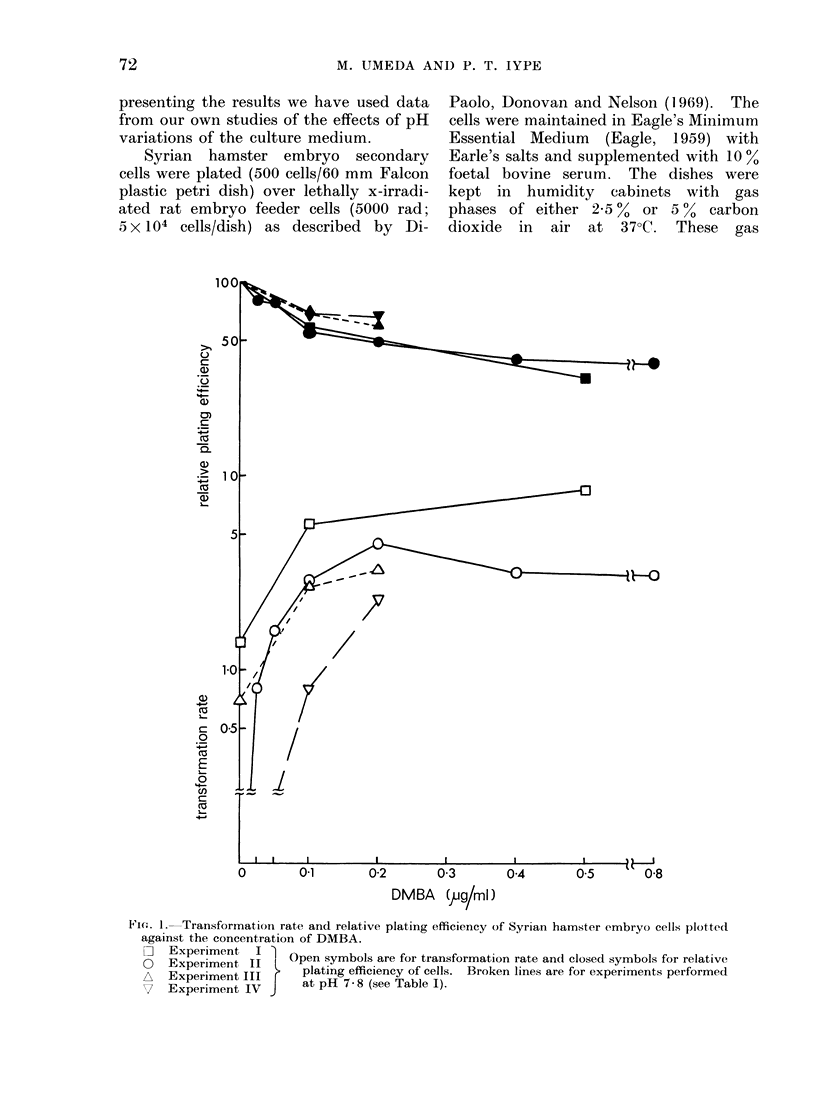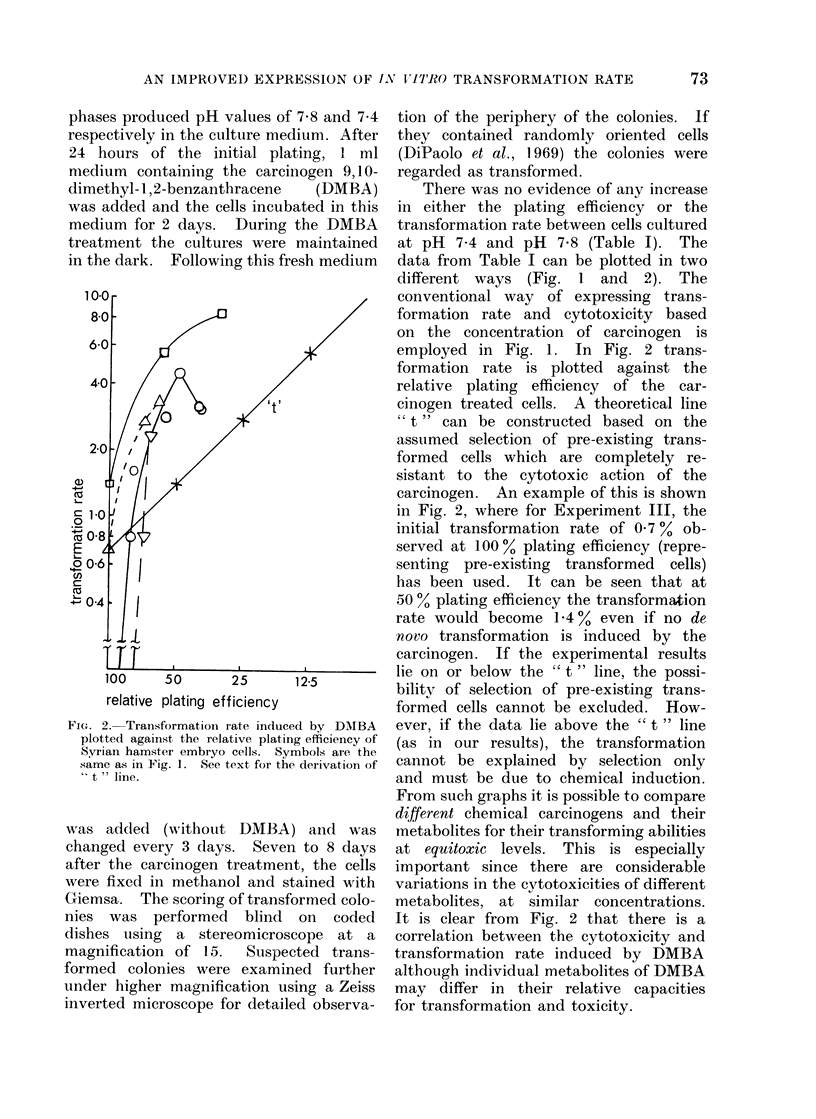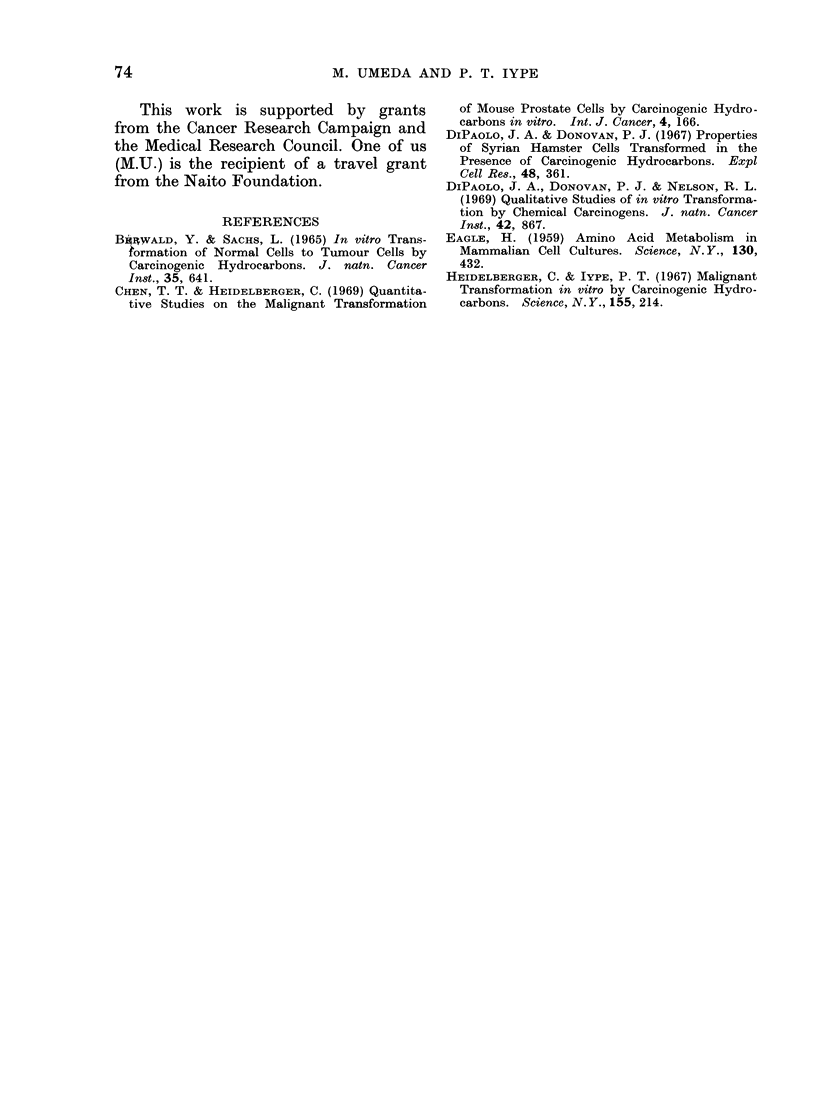# An improved expression of in vitro transformation rate based on cytotoxicity produced by chemical carcinogens.

**DOI:** 10.1038/bjc.1973.72

**Published:** 1973-07

**Authors:** M. Umeda, P. T. Iype


*Br. J. Cancer* (1973) **28**, 71

### AN IMPROVED EXPRESSION OF *IN VITRO* TRANSFORMATION RATE BASED ON CYTOTOXICITY PRODUCED BY CHEMICAL CARCINOGENS

M. UMEDA* AND P. T. IYPE

*From the Paterson Laboratories, Christie Hospital and Holt Radium Institute, Manchester M20 9BX*

Received 16 March 1973. Accepted 23 March 1973

" TRANSFORMATION " of mammalian cells in culture by carcinogenic hydrocarbons has been reported in a number of assay systems employing different cell lines, culture techniques and assessments of transformation (Berwald and Sachs, 1965; Heidelberger and Iype, 1967; DiPaolo and Donovan, 1967; Chen and Heidelberger, 1969). Almost all the chemical carcinogens tested had cytotoxic as well as transforming effects on cells *in vitro*. In such experiments, the transformation rate and survival curves were plotted independently against the concentrations of the carcinogen used. This made it difficult to compare the transformation rate at *equitoxic* levels of different carcinogens. We have overcome this problem by plotting the transformation rate against the relative plating efficiency of the carcinogen-treated cells. To compare the alternative way of

TABLE I.—*Transformation and Cytotoxicity Produced in Syrian Hamster Embryo Cells by 9,10-dimethyl-1,2-benzanthracene*

| Experiment | pH of culture medium | Concentration of DMBA (μg/ml) | No. of dishes | Total no. of colonies | Plating efficiency | No. of transformed colonies | Transformation rate (%) |
|---|---|---|---|---|---|---|---|
| I | 7·4 | 0 | 5 | 147 | 5·9 (100) | 2 | 1·4 |
| | | 0·1 | 5 | 87 | 3·5 (59) | 5 | 5·7 |
| | | 0·5 | 5 | 47 | 1·9 (32) | 4 | 8·5 |
| II | 7·4 | 0 | 30 | 1271 | 8·5 (100) | 0 | 0 |
| | | 0·025 | 15 | 513 | 6·8 (80) | 4 | 0·8 |
| | | 0·050 | 15 | 498 | 6·6 (78) | 8 | 1·6 |
| | | 0·100 | 15 | 358 | 4·8 (56) | 10 | 2·8 |
| | | 0·200 | 17 | 359 | 4·2 (49) | 16 | 4·5 |
| | | 0·400 | 18 | 309 | 3·4 (40) | 10 | 3·2 |
| | | 0·800 | 18 | 287 | 3·2 (38) | 9 | 3·1 |
| III | 7·8‡ | 0 | 15 | 559 | 7·5 (100) | 4 | 0·7 |
| | | 0·1 | 17 | 443 | 5·2 ( 69) | 12 | 2·7 |
| | | 0·2 | 15 | 338 | 4·5 (60) | 11 | 3·3 |
| IV | 7·8† | 0 | 15 | 457 | 6·1 (100) | 0 | 0 |
| | | 0·1 | 12 | 251 | 4·2 (69) | 2 | 0·8 |
| | | 0·2 | 15 | 298 | 4·0 (66) | 7 | 2·3 |

‡ The cells were maintained at pH 7·8 for 24 hours before the addition of DMBA. Carcinogen treatment and further cultivation were carried out at pH 7·4.
† The cells were maintained at pH 7·8 only during the carcinogen treatment.

* Permanent address: Tissue Culture Laboratory, Yokohama City University, Urafunecho, Minamiku, Yokohama, Japan.

presenting the results we have used data from our own studies of the effects of pH variations of the culture medium.

Syrian hamster embryo secondary cells were plated (500 cells/60 mm Falcon plastic petri dish) over lethally x-irradiated rat embryo feeder cells (5000 rad; $5 \times 10^4$ cells/dish) as described by DiPaolo, Donovan and Nelson (1969). The cells were maintained in Eagle's Minimum Essential Medium (Eagle, 1959) with Earle's salts and supplemented with 10% foetal bovine serum. The dishes were kept in humidity cabinets with gas phases of either 2·5% or 5% carbon dioxide in air at 37°C. These gas

FIG. 1.—Transformation rate and relative plating efficiency of Syrian hamster embryo cells plotted against the concentration of DMBA.

□ Experiment I

○ Experiment II

△ Experiment III

▽ Experiment IV

Open symbols are for transformation rate and closed symbols for relative plating efficiency of cells. Broken lines are for experiments performed at pH 7·8 (see Table I).

phases produced pH values of 7·8 and 7·4 respectively in the culture medium. After 24 hours of the initial plating, 1 ml medium containing the carcinogen 9,10-dimethyl-1,2-benzanthracene (DMBA) was added and the cells incubated in this medium for 2 days. During the DMBA treatment the cultures were maintained in the dark. Following this fresh medium

FIG. 2.—Transformation rate induced by DMBA plotted against the relative plating efficiency of Syrian hamster embryo cells. Symbols are the same as in Fig. 1. See text for the derivation of " t " line.

was added (without DMBA) and was changed every 3 days. Seven to 8 days after the carcinogen treatment, the cells were fixed in methanol and stained with Giemsa. The scoring of transformed colonies was performed blind on coded dishes using a stereomicroscope at a magnification of 15. Suspected transformed colonies were examined further under higher magnification using a Zeiss inverted microscope for detailed observation of the periphery of the colonies. If they contained randomly oriented cells (DiPaolo *et al.*, 1969) the colonies were regarded as transformed.

There was no evidence of any increase in either the plating efficiency or the transformation rate between cells cultured at pH 7·4 and pH 7·8 (Table I). The data from Table I can be plotted in two different ways (Fig. 1 and 2). The conventional way of expressing transformation rate and cytotoxicity based on the concentration of carcinogen is employed in Fig. 1. In Fig. 2 transformation rate is plotted against the relative plating efficiency of the carcinogen treated cells. A theoretical line " t " can be constructed based on the assumed selection of pre-existing transformed cells which are completely resistant to the cytotoxic action of the carcinogen. An example of this is shown in Fig. 2, where for Experiment III, the initial transformation rate of 0·7 % observed at 100 % plating efficiency (representing pre-existing transformed cells) has been used. It can be seen that at 50 % plating efficiency the transformation rate would become 1·4 % even if no *de novo* transformation is induced by the carcinogen. If the experimental results lie on or below the " t " line, the possibility of selection of pre-existing transformed cells cannot be excluded. However, if the data lie above the " t " line (as in our results), the transformation cannot be explained by selection only and must be due to chemical induction. From such graphs it is possible to compare *different* chemical carcinogens and their metabolites for their transforming abilities at *equitoxic* levels. This is especially important since there are considerable variations in the cytotoxicities of different metabolites, at similar concentrations. It is clear from Fig. 2 that there is a correlation between the cytotoxicity and transformation rate induced by DMBA although individual metabolites of DMBA may differ in their relative capacities for transformation and toxicity.

This work is supported by grants from the Cancer Research Campaign and the Medical Research Council. One of us (M.U.) is the recipient of a travel grant from the Naito Foundation.

### REFERENCES

BERWALD, Y. & SACHS, L. (1965) *In vitro* Transformation of Normal Cells to Tumour Cells by Carcinogenic Hydrocarbons. *J. natn. Cancer Inst.*, **35**, 641.

CHEN, T. T. & HEIDELBERGER, C. (1969) Quantitative Studies on the Malignant Transformation of Mouse Prostate Cells by Carcinogenic Hydrocarbons *in vitro*. *Int. J. Cancer*, **4**, 166.

DIPAOLO, J. A. & DONOVAN, P. J. (1967) Properties of Syrian Hamster Cells Transformed in the Presence of Carcinogenic Hydrocarbons. *Expl Cell Res.*, **48**, 361.

DIPAOLO, J. A., DONOVAN, P. J. & NELSON, R. L. (1969) Qualitative Studies of *in vitro* Transformation by Chemical Carcinogens. *J. natn. Cancer Inst.*, **42**, 867.

EAGLE, H. (1959) Amino Acid Metabolism in Mammalian Cell Cultures. *Science, N.Y.*, **130**, 432.

HEIDELBERGER, C. & IYPE, P. T. (1967) Malignant Transformation *in vitro* by Carcinogenic Hydrocarbons. *Science, N.Y.*, **155**, 214.